# One Welfare: Assessing the Effects of Drought and the COVID-19 Pandemic on Farmers’ Well-Being and Their Perception of Goats’ Welfare

**DOI:** 10.3390/ani13203297

**Published:** 2023-10-23

**Authors:** Cristian Larrondo, Raúl David Guevara, Javiera Calderón-Amor, Carolina Munoz, Carolina Cáceres, Mabeley Alvarado, Marcela Fresno, Francisca Di Pillo

**Affiliations:** 1 Núcleo de Investigaciones Aplicadas en Ciencias Veterinarias y Agronómicas, Facultad de Medicina Veterinaria y Agronomía, Universidad de Las Américas, Sede Viña del Mar, 7 Norte 1348, Viña del Mar 2531098, Chile; 2 AWEC Advisors S.L. Eureka Building. Parc de Recerca de la UAB, 08193 Cerdanyola del Valles, Spain; raul.guevara@awec.es; 3 Escuela de Graduados, Facultad de Ciencias Veterinarias, Universidad Austral de Chile, Valdivia 5090000, Chile; j.calderon.amor@gmail.com; 4 Animal Welfare Science Centre, The University of Melbourne, Melbourne, VIC 3052, Australia; munoz.c@unimelb.edu.au; 5 Facultad de Medicina Veterinaria y Agronomía, Universidad de Las Américas, Sede Viña del Mar, 7 Norte 1348, Viña del Mar 2531098, Chile; ccaceresradic@gmail.com (C.C.); mabeley.alvarado@gmail.com (M.A.); 6 Núcleo de Investigaciones Aplicadas en Ciencias Veterinarias y Agronómicas, Facultad de Medicina Veterinaria y Agronomía, Universidad de Las Américas, Sede Santiago, Manuel Montt 948, Santiago 7500972, Chile; mfresno@udla.cl (M.F.); fdipillo@udla.cl (F.D.P.)

**Keywords:** farmers’ mental health, goat farming, drought, COVID-19

## Abstract

**Simple Summary:**

The combined effects of prolonged drought with the COVID-19 pandemic have led to challenges in goat production, affecting the sustainability of farming families and goat welfare. This study aimed to investigate the impacts of these factors at the farm level and on farmers’ well-being in the Coquimbo region of Chile. This study also assessed the relationship between farmers’ perceptions of their goats’ welfare and mental health indicators. Small-scale goat farmers were interviewed using a telephone survey. They were asked about information regarding their farm and the challenges resulting from exposure to drought and the COVID-19 pandemic. The survey also included Likert scales and a questionnaire aimed at comprehending farmers’ perceptions of these events and assessing their impacts. Nearly all farmers perceived disruptions in the food supply chain, a lack of water for their animals, and economic constraints. These factors had negative effects on goat productivity, animal health, and farmers’ mental health. We found that farmers who perceived their animals to be in a good state of welfare showed better mental health indicators and were more motivated to work with them. A close link between farmers’ well-being and animal welfare was identified. Mentally healthy farmers are more likely to be attentive and proactive toward their animals, contributing to better overall welfare and farm sustainability.

**Abstract:**

Considering the interconnections between human well-being, animal welfare, and the environment, this study aimed to investigate the impacts of drought and the COVID-19 pandemic on small-scale goat farmers’ well-being and their perception of goats’ welfare following the One Welfare framework. Using a telephone survey, close-ended questions, and Likert scales, we assessed the impacts of drought and the COVID-19 pandemic on human well-being and animal welfare in the Coquimbo region of Chile. The DASS-21 questionnaire was used to evaluate farmers’ mental health. Goat farmers perceived the scarcity of water and food for animals as factors that negatively affected animal productivity and welfare and caused an increase in farmers’ stress levels. Farmers who had not been visited by a veterinarian showed higher levels of stress than those who received one visit during the year (M = 10 vs. 2, *p* = 0.025). Additionally, farmers who perceived better welfare of their animals showed lower levels of depression (*r_s_* = −0.17, *p* = 0.048), anxiety (*r_s_* = −0.21, *p* = 0.016), and stress (*r_s_* = −0.33, *p* < 0.001). These findings emphasize the importance of addressing farmers’ mental health and veterinary support as crucial aspects to ensure both goat welfare and farm productivity.

## 1. Introduction

Droughts are a natural phenomena characterized by a prolonged dry period that can significantly jeopardize the sustainability of production systems [1,2]. In Chile, it is widely acknowledged that the goat industry has been significantly impacted by the recent drought. The Coquimbo region, which is renowned as the main goat production area in the country, has been undergoing a persistent decline in rainfall of approximately 30% since 2010 [3], along with experiencing the hottest decade observed in the past century [3]. The normal average annual rainfall in this region is about 130 mm, whereas in drought conditions it is less than 30 mm [3]. The convergence of these circumstances has led to the classification of a “mega-drought,” and the region was declared to be in an agricultural emergency on 21 August 2021 [3,4]. Goat production systems in Chile are highly dependent on rainfall and other environmental factors. As expected, the drought has had a profound impact on rural potable water systems, resulting in a substantial reduction in fodder availability. This consistent reduction in both quantity and quality of available forage poses a significant threat to the welfare of goats and to the profitability and sustainability of peasant families [2,5].

Although the welfare of animals is clearly compromised under drought conditions, it is important to recognize that the mental and physical health of livestock farmers can also be affected, compromising their overall quality of life and perceptions of well-being [6]. In 2020, while goat farmers were struggling with severe drought in the Coquimbo region, the World Health Organization (WHO) officially declared the outbreak of the COVID-19 pandemic. As a result, the health authorities in Chile enforced movement restriction measures, including quarantines and lockdowns. These new circumstances resulted in disruptions within the food supply chain, which led to a rise in agricultural input prices and economic constraints in the procurement and sale of agricultural products [7]. Gradual disasters and threats, such as droughts and pandemics, can have a more detrimental and long-term impact on the environment and individuals compared to events that arise abruptly [8]. These events can result in environmental, social, and economic damage, affecting both flora and fauna [7,8], but they can also increase the appearance of depressive, anxious, and stress symptoms in individuals [9,10,11].

Clinical depression is a complex and variable mental disorder that affects approximately 280 million people in the world [12]. This disorder involves the co-occurrence of different affective, cognitive, and behavioral alterations lasting two weeks or longer [13]. According to the American Psychiatric Association, depression is characterized by more than ten symptoms, of which sad (low) mood and anhedonia (the loss of pleasure) are considered key diagnostic criteria [13]. Its diagnosis resides in the self-reporting of symptoms via interviews, questionnaires, or checklists [13]. Depression and anxiety are closely linked; not only do both show an overlap of clinical symptoms but they also share physiological changes such as dysregulation in the serotonergic and noradrenergic systems [14,15].

The concept of mental health is considered broad and complex, with multifactorial aspects. Its balance is influenced by the interaction of social, affective, economic, health, biological, psychological, and cultural elements [16]. Moreover, mental health can have serious repercussions on the well-being of the individuals and society. Popular knowledge associates farm or agricultural life with being “less stressful” or having “fewer hurries” compared to an urban lifestyle. However, farm life entails different concerns and pressures that are often overlooked from a city perspective [17]. One of the main concerns for farmers is the impact of climate change on farm production, particularly drought, as it directly affects the soil’s fertility and, consequently, animal productivity [6,18].

Since there are important interconnections between human well-being, animal welfare, and the environment, the consequences of these extreme and prolonged events should be assessed using a more holistic approach. Using the framework of One Welfare, this study investigated the effects of drought and the COVID-19 pandemic on the well-being of small-scale goat farmers. This study also assessed the relationship between farmers’ perceptions of their goats’ welfare and indicators of depression, anxiety, and stress.

## 2. Materials and Methods

The study was conducted from May to December 2021 and was approved by the Scientific Ethical Committee of the Universidad de Las Américas, Chile (CEC_FP_2023003). Two steps were involved in this cross-sectional study: (1) a pilot study to test the survey instrument and (2) a validated survey targeting goat farmers from the Coquimbo region. The Coquimbo region is located in Central Chile (between 29°20′ and 32°15′ south latitude) and its climate varies from semi-desert to Mediterranean temperate [3].

### 2.1. Pilot Study

The pilot study involved telephone surveys targeting small-scale goat farmers in the Metropolitana and Valparaíso regions of Chile. This pilot study aimed to test and validate the survey instrument. Twelve goat farmers completed the pilot survey. From these preliminary results, the survey was refined. Overall, two questions were removed and three questions were reworded to improve clarity.

### 2.2. Survey of Goat Farmers

#### 2.2.1. Participants

Following the completion of the pilot study, a comprehensive database of 267 goat farmers was obtained through a collaboration with government institutions associated with the Ministry of Agriculture of Chile. These farmers were previously enrolled in the Agricultural and Livestock Program for the Integral Development of Small Dryland Farmers in the Coquimbo region (PADIS). The final version of the survey was applied via telephone and a total of 130 goat farmers were contacted, agreed to participate, and completed the survey.

#### 2.2.2. The Survey Instrument

The survey was designed following a One Welfare approach, meaning that a combination of questions addressing the impacts of drought and the COVID-19 pandemic on human well-being and animal welfare were included. The survey questions were administered via telephone interviews (Survey S1), and data were collected using the Google Forms^®^ platform. The study was voluntary and ensured anonymity and confidentiality. If respondents declined to participate at the beginning or during the survey, they were thanked for their time and the call was concluded.

The survey comprised two sections and a total of 49 questions. The estimated duration of the survey was 20 min. The first section focused on demographic and general aspects of the production system, including questions related to the participant’s gender, age (in years), the type of ruminants present on their farms, the specific purpose of their production (e.g., dairy goats), the management approach employed in their production system (extensive, semi-intensive, or intensive), and the total number of animals. Additionally, farmers were asked whether their farms had received visits from a veterinarian during the current year, and if so, the frequency of those visits was recorded (monthly, every three months, only once, or never).

The second section of the survey focused on gathering information about farmers’ perceptions and challenges resulting from drought and the COVID-19 pandemic. Using closed-ended questions with a binary response format (Yes/No), farmers were asked about the impact of these events on animal husbandry, particularly on animal health, feed and water access, feed costs, and mortality rates, as well as the need to sell/relocate or euthanize animals. Participants were also directly asked whether they believed that COVID-19 could impact their livestock, whether they had to undergo quarantine during this period, and whether the situation had a detrimental effect on their work with their animals.

Likert-scale questions were also employed to evaluate farmers’ perceptions regarding the impact of drought and the pandemic on various factors throughout 2021. These factors included the productivity of their animals, economic resources, animal welfare, and whether they had experienced a sense of discouragement in working with their animals.

#### 2.2.3. DASS-21 Questionnaire

Lastly, the DASS-21 questionnaire was used [19], which has been previously utilized in studies to assess the mental health of farmers [20,21,22] and to evaluate the impact of COVID-19 on diverse populations [23,24]. The DASS-21 is a self-report questionnaire consisting of a total of 21 items (Questionnaire S1). This questionnaire facilitated the assessment of whether farmers had encountered circumstances associated with symptoms of depression, anxiety, or stress in the week preceding the survey. Each item was scored on a scale of 0 to 3 based on the occurrence of the corresponding statement (0 = Did not apply to me at all; 1 = Applied to me to some degree, or some of the time; 2 = Applied to me to a considerable degree or a good part of the time; 3 = Applied to me very much or most of the time). The final results were obtained after adding the total score and multiplying it by two [19]. The final scores were classified into levels as follows: depression: normal (0–9), mild (10–13), moderate (14–20), severe (21–27), and extremely severe (28 and above); anxiety: normal (0–7), mild (8–9), moderate (10–14), severe (15–19), and extremely severe (20 and above); and stress: normal (0–14), mild (15–18), moderate (19–25), severe (26–33), and extremely severe (34 and above).

### 2.3. Data Management and Statistical Analyses

The demographic data, perceptions of animal welfare, individual well-being, and scores obtained from the DASS-21 questionnaire were summarized using descriptive statistics. Generalized Poisson regression models (*p* < 0.05) were used to assess the impact of drought and the COVID-19 pandemic on farmers’ well-being and goats’ welfare, using scores obtained from the DASS-21 subscales (depression, anxiety, and stress) as dependent variables and demographic and perception questions as independent variables. Likert-scale questions were also used as covariables in these regression models.

The internal consistency of the DASS-21 questionnaire and its subscales was calculated using the Cronbach’s alpha statistic. In addition, Spearman correlations were utilized (*p* < 0.05) to assess the relationship between levels of depression, anxiety, and stress, as well as farmers’ perceptions of the impact of drought and COVID-19 on their well-being and goat welfare. Statistical analyses for this study were performed using the SPSS version 26 software (SPSS Inc., Chicago, IL, USA).

## 3. Results

### 3.1. Demographic Information of the Goat Farmers and Their Farms

The final response rate of the telephone survey was 48.69% (130 farmers out of 267 were successfully contacted and agreed to participate in the study). Most of the respondents were men (*n* = 73; 56.15%) and the rest were women (*n =* 57; 43.85%), with a median age of 55.5 years (IQR: 46–64). Twenty-three farmers (29.9%) had other ruminants (sheep and/or cattle) in addition to goats on the farm. The median number of goats in each system was 44 animals (IQR: 30–65), mostly managed under semi-intensive systems (partial access to pastures; 63.08%), followed by intensive (animals kept indoors in pens; 26.15%) and extensive (natural pasture-based feeding systems; 10.77%) management. Most survey respondents (49%) managed dual-purpose goats (meat and milk), followed by dairy goats (46%) and meat-focused goats (5%).

Regarding the frequency of veterinary visits in 2021, considering both preventive care and treatment, nearly half of the farmers (46.92%) reported not having received any veterinary care. Meanwhile, 22.31% of farmers indicated that they had been visited by a veterinarian at least once during the year, with 16.15% reporting visits every three months and 14.62% reporting monthly visits.

### 3.2. Impacts of Drought and COVID-19 Pandemic on the Well-Being of Goat Farmers and Their Animals

Table 1 shows the results from questions related to the impact of drought and COVID-19 on farm management. The majority of the farmers surveyed (83.08%) indicated that the ongoing drought and pandemic resulted in adverse economic consequences for them, specifically impacting their ability to secure animal feed. Nearly all respondents (97.69%) perceived a notable increase in the costs associated with these inputs compared to the previous year. A total of 76.92% of farmers reported a limited water supply for their animals, with 42.31% reporting that animals had died on their farms due to a lack of water/food availability.

**Figure 1:**
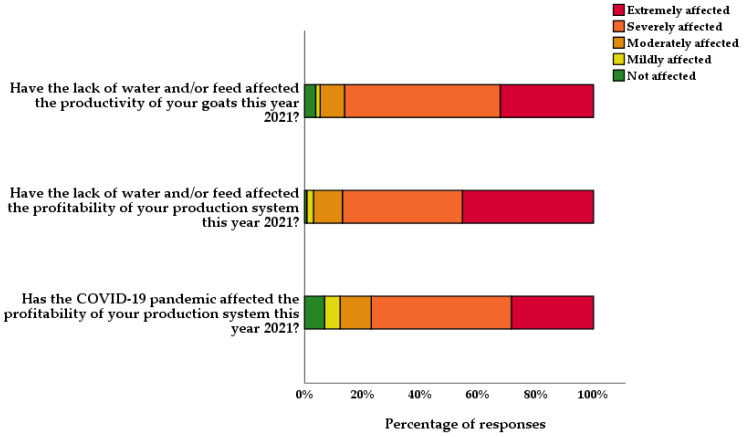
Bar graph showing the impacts of drought and the COVID-19 pandemic on goats’ productivity and farm profitability during the year 2021. Scores from the Likert scale (0–7) were categorized as follows: not affected (0), mildly affected (1), moderately affected (2–3), severely affected (4–5), and extremely affected (6–7).

Figure 1 illustrates farmers’ perceptions of the impacts of drought and the COVID-19 pandemic on goats’ productivity and farm profitability. This was measured using a Likert scale ranging from 0 (no impact) to 7 (significant impact). A total of 112 out of 130 goat farmers (82.15%) perceived that the lack of water and/or food was a factor that negatively affected the animals’ productivity and yielded scores of 4 or more (Figure 1).

Farmers’ perceptions of animal welfare resulted in a mode and median score of 5, with the scoring scale ranging from 0 (very bad) to 7 (very good). Similarly, when asked about feelings of discouragement towards working with their animals (Survey S1), the most common score was 5, with a median score of 4. A score of 0 indicated no feelings of discouragement, whereas a score of 7 indicated many feelings of discouragement.

### 3.3. DASS-21 and Its Relationship with the Demographic Variables of Goat Farmers

The DASS-21 questionnaire presented good levels of internal consistency based on an α coefficient of 0.87. Significant positive correlations were observed among the scores obtained in each DASS-21 subscale. Specifically, there were positive correlations between levels of depression and anxiety (*r_s_* = 0.60, *p* < 0.05), depression and stress (*r_s_* = 0.69, *p* < 0.05), and anxiety and stress (*r_s_* = 0.65; *p* < 0.05). In addition, the depression, anxiety, and stress subscales exhibited α coefficients of 0.83, 0.82, and 0.80, respectively.

Table 2 presents the distribution of depression, anxiety, and stress levels, categorized by severity and stratified by gender. Based on the scores obtained from the DASS-21 subscales, a total of 19 (14.62%) farmers out of the 130 that participated in the survey reported signs of depression, 32 (24.62%) reported signs of anxiety, and 21 (16.15%) reported signs of stress, ranging from mild to extremely severe. Gender was the only sociodemographic variable in which significant differences were found in the DASS-21 subscales. Overall, female participants reported higher levels of anxiety (31.58%) compared to male participants (19.18%), with M = 4.00 vs. 2.00, respectively and *p* = 0.019 (Table 2).

### 3.4. DASS-21 and Its Relationship with the Impacts of Drought and COVID-19 Pandemic

**Figure 2:**
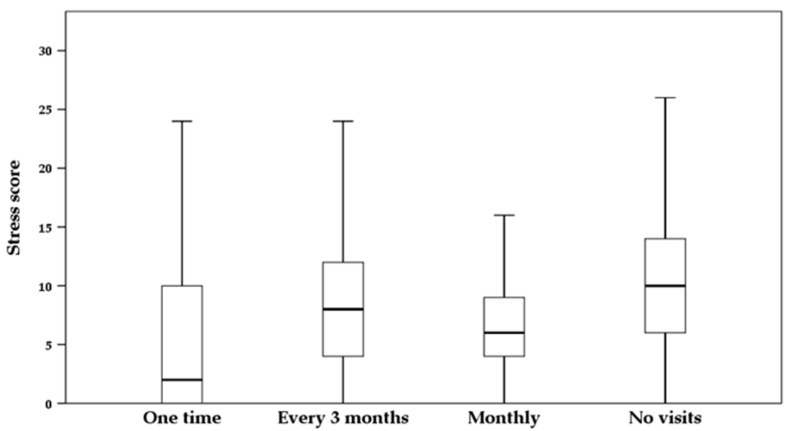
Box-and-whisker plot showing the stress score levels of goat farmers from the Coquimbo region in relation to the frequency of veterinary visits to their production system during the year 2021. Results obtained from the DASS-21 stress subscale.

Significant relationships were observed between the frequency of veterinary visits and the DASS-21 stress scores (χ^2^(3) = 8.676, *p* = 0.034) (Figure 2). Farmers who reported zero visits to their farm in 2021 had a median score of 10.00 in the DASS-21 stress scores, whereas those who reported only one visit had a median score of 2.00 (χ^2^(3) = 1187.500, *p* = 0.025). The stress scores ranged from 0 (low) to 28 (high).

There were several factors that significantly affected farmers’ stress levels, such as economic problems for buying food for their animals (*p* < 0.001), their perception of an increase in the cost of animal feed (*p* = 0.005), the possibility that the lack of water and/or feed affected the health of their animals (*p* < 0.001), the need to sell animals due to a lack of water and/or feed (*p* = 0.001), and other factors, as shown in Table 3.

The need to relocate or sell animals due to a lack of feed and/or water were variables that significantly predicted the levels of anxiety and stress (Table 3). Additionally, farmers’ perception that the lack of water and/or feed affected the productivity of the goats predicted the levels of depression (*p* = 0.038) and anxiety (*p* = 0.002; Table 3). This perception was negatively associated with farmers’ stress levels (*r_s_* = 0.18, *p* = 0.032).

Farmers’ feeling of discouragement about working with their animals and their perception of their goats’ welfare were factors that significantly affected the three DASS-21 subscales (*p* < 0.001; Table 3). A positive correlation was also found between the perceived lack of motivation to work with their animals and the levels of anxiety (*r_s_* = 0.24, *p* = 0.005) and stress (*r_s_* = 0.23, *p* = 0.008) among farmers. Negative correlations were observed between perceived levels of animal welfare and the DASS-21 subscales: depression (*r_s_* = −0.17, *p* = 0.048), anxiety (*r_s_* = −0.21, *p* = 0.016), and stress (*r_s_* = −0.33, *p* < 0.001). This indicates that farmers who perceived higher levels of welfare in their animals exhibited fewer signs of depression, anxiety, and stress.

## 4. Discussion

The recent emergence of zoonotic diseases such as COVID-19, Ebola, and avian influenza has underscored the critical importance of recognizing the interconnections between human, animal, and environmental health [25,26]. Addressing the root causes of these diseases requires collaborative efforts between medical professionals, veterinarians, ecologists, public health experts, and policymakers to identify and implement effective control measures. The One Health approach advocates for precisely this interdisciplinary cooperation, acknowledging the inherent linkages between the health of humans, animals, and the environment [27,28].

Addressing the complex interactions of zoonotic diseases and other health challenges across species requires embracing another fundamental concept known as One Welfare. One Welfare extends the One Health approach by considering the well-being of not only animals and humans but also the broader ecological and social context. It recognizes the deep interconnectedness between the welfare of animals, humans, and the environment, highlighting that improving one aspect of welfare can positively influence the others [29]. An excellent example of this interconnectedness is how enhancing animal welfare on farms can lead to multiple positive effects on human health. Not only does it help reduce the risk of zoonotic diseases, but it also improves food safety and food security and even promotes better mental health for farmers [30]. Looking ahead to the year 2050, the global population is projected to exceed 9 billion people, posing a significant challenge in terms of feeding the world. Agriculture and livestock will play a crucial role in meeting this demand [31,32]. It is now well known that optimal animal production is achieved when animals are in their best state of health [33]. However, for animals to be in optimal health, their caretakers (farmers) must also be in good health, including mental health, to provide the best care for their animals [34].

The effects of drought on goat productivity have a negative impact on farm profitability, directly affecting the quality of life of farmers who heavily rely on agricultural production to support their families [35]. Goats are known for their adaptability to drought conditions and their ability to maintain their productivity even under extreme climatic conditions [36,37] in arid and semi-arid ecosystems [38]. However, severe limitations in foraging and water can significantly impact their productivity and welfare [37,39]. This concern was reported by 112 out of 130 goat farmers who participated in this study, where the lack of water and/or food was perceived to be a factor that negatively affected the animals’ productivity and yielded scores of 4 or more on a scale from 0 to 7. These findings are in agreement with other studies, which have reported that low animal production can have an impact on the mental health of farmers. A systematic review by Yazd et al. [40] identified key risk factors affecting farmers’ mental health, including financial difficulties, climate variabilities/drought, and poor physical health.

Farm profitability is one of the main concerns of the agricultural population, and it is closely related to farmers’ deteriorating mental health [17,41]. Australian researchers have reported that diminished mental health can be associated with the impact of extreme climate conditions [35]. Additionally, due to this persistent worry, farmers’ mental health is likely to be vulnerable due to factors such as the lack of availability of mental health services in nearby rural communities, the “stoic” culture of farmers avoiding acknowledging mental health vulnerability and seeking proper assistance, and the fragility of the family economy [18,41,42]. Despite the increasing trend of raising awareness about mental health, few studies on farmers’ mental health have been published [30,43]. However, farmers’ mental health should be a public concern, as they are a key factor in agriculture production to satisfy the feeding requirements of a growing society, and they play a central role in the implementation of animal welfare-friendly practices [43].

The COVID-19 pandemic has also had a negative impact on the mental health of farmers globally [44] and a tremendous impact on human society not only in terms of health but also economically and in terms of humanitarian aspects, triggering a decrease in employment, reductions in family income, and general uncertainty for companies and industries [10,11,45]. Goat farms, being small family businesses, were highly vulnerable to the pandemic’s effects, leading to reduced commerce, decreased family income, and heightened stress levels among farmers. Moreover, farmers already faced concerns about providing water for the goats and maintaining grasslands due to drought [6,18,46]. Survey participants stated that the pandemic restrictions limited access to essential supplies, transportation, and veterinary services. Veterinary support seems to play an important role in farmers’ mental health during times of uncertainty. In this study, farmers who reported not having received veterinary assistance for their animals showed higher levels of stress than those who received only one visit during the year. These results align with those previously reported in the same region [47], reflecting the farmers’ concern with ensuring the welfare of their goats, the importance of veterinary assistance in a scenario of uncertainty and the need to address the effects of drought and the pandemic under the One Health and One Welfare approaches.

Access to food and water were the main concerns for goat farmers, clearly affecting their management decisions and stress levels. A total of 10% of the survey participants reported moderate levels of anxiety. These results are a bit higher than reports by Mishra and Satapathy [48], who assessed the incidence of depression, anxiety, and/or stress among agricultural farmers in India during the pandemic. In the present study, farmers that reported any level of depression (14.62%), anxiety (24.62%), or stress (16.15%) were associated with difficulties with providing feed or water to their animals. Further, farmers that reported dead animals due to lack of feed and water were even more stressed (moderate to severe). Even if the reported stress levels were relatively low, the strong dependence of family income on farm production (soil fertility, animal growth, and production) can increase the incidence of depression, anxiety, and stress [20,21,22].

The difficulties reported by goat farmers in terms of providing food and water to their goats had negative impacts on their farms, leading them to have to change certain husbandry practices, such as reducing the herd through sales or slaughtering the animals, and to adapt their production system to the new economic situation. These management decisions were also reported by livestock farmers in Argentina, Bolivia, Paraguay, Peru, and the Dominican Republic [7]. Farm management decisions, such as reduction in herd size, are likely to affect farmers’ profits, with direct impacts on the family income, which will increase farmers’ mental pressure. Griffith et al. [49] and FAO [50] reported that sheep farmers face difficulties with taking sheep to graze due to transhumance restrictions during the COVID-19 lockdowns and quarantines, which made even more serious the pressure/distress of feeding sheep.

Farmers worldwide are facing several stressors, such as climate change and economic changes in agriculture [18], and have a higher probability of developing mental health disorders than other occupational groups [18,41]. In general, women are more likely to develop stress and anxiety than men [43,51,52]. In this study, female farmers reported higher levels of anxiety than males (31.58% vs. 19.18%, respectively). These results are similar to results reported in the UK [52], Australia [53], and Canada [43]. Women have an important role in rural communities, and due to the changing dynamics of farm work, they have to face several challenges, such as role conflicts and high workloads. The impact on family well-being is a significant factor contributing to the stress experienced by women in the agricultural sector [54].

Remarkably, the results from this survey revealed a significant relationship between farmers’ mental health (depression, anxiety, or stress) and their perception of their animals’ welfare. Farmers who perceived good animal welfare were less likely to experience depression, anxiety, or stress. The effect and relationship observed in this study between farmers’ mental health and their goats’ welfare could indicate that farmers are likely to project their own well-being onto their animals, highlighting the importance of addressing mental health to promote better animal welfare outcomes [55,56]. In addition, the experience of depression, anxiety, or stress by goat farmers negatively impacted their motivation to work with the animals and could tend to reduce animal welfare-friendly practices. Farmers experiencing depression, anxiety, or stress exhibit lower levels of attention and care towards their animals, which compromises their engagement with the proper performance of good handling practices [43]. On the other hand, farmers with better mental health are more attentive, proactive, and empathetic towards their animals, promoting better animal welfare outcomes. Positive attitudes of farmers towards working with their animals are associated with positive animal welfare outcomes [57]; therefore, improving farmers’ mental health could be a strategy to preserve animal welfare. In addition, it has to be acknowledged that farmers’ mental health is highly vulnerable due to different cultural factors.

## 5. Conclusions

Recent health challenges have emphasized the need for a comprehensive approach that considers the connections between human, animal, and environmental health. The One Health approach has been pivotal in fostering interdisciplinary collaboration, but it must be complemented by the One Welfare concept to address the complex interactions across species. The results from this study indicate that animal health is linked to the mental health of farmers, highlighting the need to increase awareness and support. Furthermore, veterinarians seem to play an important role, helping farmers to feel less stressed and anxious during uncertain periods. Farmers’ mental health can be highly vulnerable due to economic, cultural, and environmental factors and limited access to mental health support in rural areas. Environmental concerns such as drought have been one of the main stressors for agricultural stakeholders, along with the COVID-19 pandemic, providing an additional stress factor that has generated new obstacles for farmers and increased uncertainty regarding farm performance and family income. These situations trigger depression, stress, and/or anxiety among farmers, particularly among females. Motivation to work with animals is also associated with the mental state of farmers. As we look toward the future, feeding the growing global population will require sustainable agriculture and livestock practices, and a focus on both animal welfare and farmers’ well-being will be vital in achieving this goal. Therefore, by applying the One Welfare approach in animal welfare and production studies, we can increase the awareness and opportunities for proper care of the mental health of livestock stakeholders and preserve animal welfare.

## Figures and Tables

**Table 1:** The impacts of drought and the COVID-19 pandemic on farmers’ perceptions of their well-being, goats’ welfare, and husbandry practices. Responses from 130 goat farmers from the Coquimbo region of Chile during the year 2021.

	Answer % (*n*)	
Question	Yes	No
Have you had economic problems buying food for the animals?	83.08 (108)	16.92 (22)
Has there been a perceived increase in the cost of animal feed compared to last year?	97.69 (127)	2.31 (3)
Has it been more difficult than last year to obtain water for the animals?	76.92 (100)	23.08 (30)
Do you think that the lack of water and/or feed has affected the health of your animals?	62.31 (81)	37.69 (49)
Have animals died on your farm due to lack of water and/or feed?	42.31 (55)	57.69 (75)
Have you had to relocate (move your animals to another farm or region) due to lack of feed and/or water?	28.46 (37)	71.54 (93)
Have you had to sell animals due to a lack of water and/or food?	61.54 (80)	38.46 (50)
Have you had to slaughter/eliminate (euthanize, shoot, slit, or otherwise kill) animals due to a lack of food and/or water?	13.08 (17)	86.92 (113)
Have you had to sell animals as a result of the COVID-19 pandemic?	38.46 (50)	61.54 (80)
Do you think that COVID-19 could affect the health of your goats, sheep, or cows?	30.00 (39)	70.00 (91)
Have you had to stay at home (quarantine) due to the COVID-19 pandemic?	73.85 (96)	26.15 (34)
If you have had to quarantine, do you think that situation has negatively affected your work with your animals?	79.63 (86)	20.37 (22)

**Table 2:** Distribution of depression, anxiety, and stress levels from the DASS-21 scale, categorized by severity and gender of the goat farmers (*n =* 130) from the Coquimbo region of Chile during the year 2021.

Variable	Male (*n =* 73)		Female (*n =* 57)		*p*-Value	Overall (*n* = 130)	
	*n*	%	*n*	%		*n*	%
**Depression level**							
Normal	64	87.67	47	82.46		111	85.38
Mild	3	4.11	7	12.28		10	7.69
Moderate	4	5.48	3	5.26		7	5.38
Severe	1	1.37	0	0.00		1	0.77
Extremely severe	1	1.37	0	0.00		1	0.77
Overall depression level	9	12.33	10	17.54	0.075	19	14.62
**Anxiety level**							
Normal	59	80.82	39	68.42		98	75.38
Mild	5	6.85	3	5.26		8	6.15
Moderate	4	5.48	9	15.79		13	10.00
Severe	2	2.74	4	7.02		6	4.62
Extremely severe	3	4.11	2	3.51		5	3.85
Overall anxiety level	14	19.18	18	31.58	0.019	32	24.62
**Stress level**							
Normal	64	87.67	45	78.95		109	83.85
Mild	5	6.85	8	14.04		13	10.00
Moderate	3	4.11	2	3.51		5	3.85
Severe	1	1.37	2	3.51		3	2.31
Extremely severe	0	0.00	0	0.00		0	0.00
Overall stress level	9	12.33	12	21.05	0.063	21	16.15

**Table 3:** Effects of drought and the COVID-19 pandemic on goat farmers’ (*n* =130) depression, anxiety, and stress levels (DASS-21 subscales) during the year 2021. Results from the regression models. Regression coefficient (β), confidence intervals for β (95% CI), and *p*-values (Wald-χ^2^).

		Depression			Anxiety			Stress		
Variable		β	CI for β	*p*-Value	β	CI for β	*p*-Value	β	CI for β	*p*-Value
Have you had economic problems buying food for the animals?	Yes	1.209	4.74–3.083	0.69	2.255	0.802–6.336	0.123	23.691	6.884–81.534	<0.001
Has there been a perceived increase in the cost of animal feed compared to last year?	Yes	6.336	1.846–21.748	0.003	18.748	3.724–94.379	<0.001	39.743	2.954–534.642	0.005
Do you think that the lack of water and/or feed has affected the health of your animals?	Yes	2.562	1.215–5.400	0.013	1.836	0.810–4.164	0.146	9.926	3.614–27.266	<0.001
Have you had to relocate your animals due to lack of feed and/or water?	Yes	4.705	1.905–11.623	0.001	3.964	1.547–10.161	0.004	3.648	1.121–11.876	0.032
Have you had to sell animals due to a lack of water and/or food?	Yes	1.840	0.846–4.001	0.124	3.989	1.743–9.129	0.001	5.802	2.080–16.182	0.001
Do you think that COVID-19 could affect the health of your goats, sheep, or cows?	Yes	1.346	0.596–3.039	0.475	1.381	0.572–3.339	0.473	2.893	0.943–8.874	0.063
Have you had to stay at home (quarantine) due to the COVID-19 pandemic?	Yes	0.862	0.363–2.045	0.736	0.935	0.376–2.323	0.885	3.176	1.043–9.672	0.042
**Covariable (Likert scales)**										
Has the lack of water and/or feed affected the productivity of your goats?		0.798	0.644–0.988	0.038	1.44	1.149–1.813	0.002	1.291	0.955–1.744	0.097
Has the lack of water and/or feed affected the profitability of your production system?		0.924	0.701–1.217	0.573	0.794	0.614–1.026	0.078	0.799	0.544–1.116	0.173
Has the COVID-19 pandemic affected the profitability of your production system?		1.180	1.004–1.386	0.44	0.837	0.690–1.016	0.072	1.310	1.020–1.682	0.035
Have you felt discouraged about working with your animals?		1.725	1.509–1.971	<0.001	1.947	1.683–2.253	<0.001	1.763	1.449–2.146	<0.001
What level of welfare do you think your animals have?		0.587	0.465–0.741	<0.001	0.611	0.482–0.776	<0.001	0.345	0.253–0.471	<0.001

## Data Availability

Data are unavailable due to privacy or ethical restrictions.

## Supplementary Materials

The following supporting information can be downloaded at: https://www.mdpi.com/article/10.3390/ani13203297/s1, Survey S1: Survey of goat farmers in Chile; Questionnaire S1: DASS-21 questionnaire.
