# Supplementary material for: One Welfare: Assessing the Effects of Drought and the COVID-19 Pandemic on Farmers’ Well-Being and Their Perception of Goats’ Welfare
Source: Animals (Basel). 2023 Oct 23;13(20):3297. doi: 10.3390/ani13203297 (PMC10603839; doi:10.3390/ani13203297)
Supplement: Supplementary file 1 [file animals-13-03297-s001.zip › Survey S1.pdf]

Survey

You have been invited to participate in a survey of goat farmers in Chile. This survey consists of three sections and is estimated to take approximately 20 minutes to complete. This survey aimed to investigated the effects of drought and the COVID-19 pandemic on your well-being and your goats.

Your participation is voluntary and confidential. The results obtained will be used for academic and research purposes. We sincerely appreciate your willingness to take part in this survey, which will provide valuable information about both human well-being and animal welfare. For any inquiries, please contact Cristian Larrondo (clarrondo@udla.cl), Veterinarian, Academic at the Faculty of Veterinary Medicine and Agronomy, Universidad de Las Américas, Chile.

Are you in agreement and willing to participate voluntarily in this study? YES/NO

I. General Information
1. Gender: Male, Female, Non-binary, I don't want to answer

2. Age (years):

4. Type of livestock production (you can select more than one): Meat Goats, Dairy Goats, Meat Sheep, Wool Sheep, Dairy Sheep, Dairy Cattle, Meat Cattle.

5. How many animals do you have? Number of: goats and sheep and/or cows if you have.

7. Have there been visits by a Veterinarian during this year 2021?
   a) Once b) Monthly c) Every 3 months d) No

II. Demographic and general aspects of the production system
1. During this year 2021, have you had economic difficulties in purchasing feed for the animals? YES/NO

2. During this year 2021, have you had problems acquiring or buying feed for the animals, due to supplier issues, logistics, or mobility? YES/NO

3. During this year 2021, has the cost of animal feed increased compared to last year? YES/NO

4. During this year 2021, has it been more difficult than the previous year to obtain water for the animals? YES/NO

5. Has drought affected the health of your animals this year 2021? YES/NO

6. Has drought affected the productivity of your animals (e.g. lower milk, and/or meat production) this year 2021? From 0 to 7, where 0 indicates no impact and 7 indicates a significant impact.

7. Has drought had an economic impact on your production system? From 0 to 7, where 0 indicates no impact and 7 indicates a significant impact.

8. Have animals on your property died due to lack of water and/or feed this year 2021?
a) No b) Less than 10 animals c) Between 11 and 50 animals d) Between 51 and 100 animals e) More than 100 animals

9. Have you had to relocate (move your animals to another property or region) due to drought this year 2021? YES/NO

10. Have you had to sell animals due to drought this year 2021? YES/NO

11. Have you had to euthanize/eliminate (euthanasia, shooting, slaughter, or other method to end life) animals due to drought? YES/NO

12. Has the Coronavirus (Covid-19) pandemic had an economic impact on your production system? From 0 to 7, where 0 indicates no impact and 7 indicates a significant impact. 0 to 7

13. Have you had to sell animals as a result of the Coronavirus (Covid-19) pandemic? YES/NO

14. Have you had to stay at home (quarantine) due to the coronavirus pandemic? YES/NO

15. If the previous response was YES, do you believe this situation has affected your work with animals? YES/NO . If the previous response was NO, leave this question blank.

16. Do you think that the coronavirus (Covid-19) can affect the health of your goats? YES/NO

17. What has affected you more this year 2021, drought or the coronavirus pandemic?
a) Drought b) Coronavirus pandemic c) Both equally d) Neither of the two e) Other event______

18. How discouraged have you felt while working with your animals in this year 2021? From 0 to 7, where a score of 0 indicated no feelings of discouragement while a score of 7 indicated a lot of feelings of discouragement.

III. DASS-21 questionnaire (See supplementary material "Questionnaire S2 DASS-21")
